# Concentrations and kinetics of renal biomarkers in dogs with gastric dilatation-volvulus with and without 24-h intravenous lidocaine

**DOI:** 10.3389/fvets.2023.1115783

**Published:** 2023-02-10

**Authors:** Anna Lehmann, Anna Brunner, Eliane Marti, Thierry Francey, Sarah Steinbach, Laureen M. Peters, Katja-Nicole Adamik

**Affiliations:** ^1^Division of Small Animal Internal Medicine, Department of Clinical Veterinary Science, Vetsuisse Faculty, University of Bern, Bern, Switzerland; ^2^Division of Small Animal Surgery, Department of Clinical Veterinary Science, Vetsuisse Faculty, University of Bern, Bern, Switzerland; ^3^Department of Clinical Research and Veterinary Public Health, Vetsuisse Faculty, University of Bern, Bern, Switzerland; ^4^Department of Veterinary Clinical Science, College of Veterinary Medicine, Purdue University, West Lafayette, IN, United States; ^5^Clinical Diagnostic Laboratory, Department of Clinical Veterinary Science, Vetsuisse Faculty, University of Bern, Bern, Switzerland; ^6^Division of Small Animal Emergency and Critical Care, Department of Clinical Veterinary Science, Vetsuisse Faculty, University of Bern, Bern, Switzerland

**Keywords:** AKI, renal biomarker, canine gastric torsion, ischemia-reperfusion, organoprotection, lidocaine

## Abstract

**Background:**

Gastric dilatation volvulus (GDV) can lead to organ failure including acute kidney injury (AKI). Due to its cytoprotective, antioxidant and anti-inflammatory effects, lidocaine has a potential to prevent AKI in dogs with GDV.

**Design and setting:**

Prospective, observational cohort study in client-owned dogs with GDV.

**Objective:**

To determine concentrations of renal biomarkers for AKI in dogs with GDV with and without intravenous (IV) lidocaine therapy.

**Methods:**

Thirty-two dogs were randomized to receive either IV lidocaine (2 mg/kg, followed by a lidocaine constant rate infusion at a dose of 50 μg/kg/min over 24 h; *n* = 17) or no lidocaine (*n* = 15). Blood and urine samples were taken at admission (*T*_0_) (only blood), during or immediately after surgery (*T*_1_), and 24 (*T*_24_) and 48 (*T*_48_) h after surgery. Plasma creatinine (pCr), plasma neutrophil gelatinase-associated lipocalin (pNGAL), urinary NGAL (uNGAL), uNGAL to creatinine ratio (UNCR), and urinary gamma-glutamyl transferase to creatinine ratio (uGGT/uCr) were evaluated. Biomarker concentrations were compared between dogs with and without IV lidocaine and the course of each marker was determined in comparison to its admission value.

**Results:**

In the entire population, a significantly higher pCr at *T*_0_ (median, 95 μmol/L, interquartile range, 82–105) compared with *T*_1_ (69 μmol/L, 60–78), *T*_24_ (63 μmol/L, 52–78), and *T*_48_ (78 μmol/L, 65–87) (*P* < 0.001) was found. Plasma NGAL increased significantly between *T*_0_ (5.66 ng/mL, 3.58–7.43) and *T*_24_ (7.50 ng/mL, 4.01–11.89) (*P* = 0.006) and *T*_48_ (9.86 ng/mL, 5.52–13.92) (*P* < 0.001), respectively. Urinary NGAL increased significantly between *T*_1_ (0.61 ng/mL, 0.30–2.59) and *T*_24_ (2.62 ng/mL, 1.86–10.92) (*P* = 0.001) and *T*_48_ (4.79 ng/mL, 1.96–34.97 (*P* < 0.001), respectively. UNCR increased significantly between *T*_1_ (0.15 μg/mmol, 0.09–0.54) and *T*_24_ (1.14 μg/mmol, 0.41–3.58) (*P* = 0.0015) and *T*_48_ (1.34 μg/mmol, 0.30–7.42) (*P* < 0.001), respectively. Concentrations of uGGT/uCr increased significantly from *T*_0_ highest at *T*_24_ (6.20 U/mmol, 3.90–9.90) and significantly decreased at *T*_48_ (3.76 U/mmol, 2.84–6.22) (*P* < 0.001). No significant differences in any renal biomarker concentration were found between dogs with and without IV lidocaine therapy.

**Conclusion and clinical relevance:**

Plasma NGAL, uNGAL and UNCR remained increased up to 48 h post-surgery. No evidence of lidocaine-associated renoprotection was found.

## Introduction

Gastric dilatation volvulus (GDV) is an acute and life-threatening condition in large breed dogs. Common consequences are impaired gastric blood supply, obstructive and distributive shock, decreased systemic tissue perfusion and ischemia (1, 2). The most severe complications associated with GDV are ischemic reperfusion-injury (IRI), systemic inflammatory response syndrome (SIRS), and multiple organ dysfunction syndrome (MODS), including cardiac arrhythmias and acute kidney injury (AKI) (2–9). AKI, detected by creatinine-based criteria, has been found to occur in 2.3 and 8% of dogs with GDV, and has been reported to be a significant risk factor for death (3, 8).

Lidocaine is a potent local anesthetic and class Ib antiarrhythmic agent. It also acts as a scavenger of reactive oxygen species and as an inflammatory modulator in both human and veterinary patients and counteracts several pathways in IRI (10, 11). In dogs with experimentally induced gastric dilation, lidocaine treatment was cardio- and gastroprotective and reduced gastric tissue damage (12). Bruchim et al. (3) found a significant decrease in the incidence of AKI and cardiac arrhythmias, and in length of hospitalization in GDV dogs receiving early intravenous (IV) lidocaine, followed by a constant rate infusion (CRI) over 24 h. However, no randomized-controlled trial has evaluated the renoprotective effect of IV lidocaine in GDV dogs.

Neutrophil gelatinase-associated lipocalin (NGAL), a protein expressed during proximal tubular epithelial cell injury, is used as a biomarker in blood and urine to detect early-stage AKI (13–16). NGAL has been found to be one of the earliest and most consistently generated proteins in ischemic and nephrotoxic AKI in human and dog models (17, 18). Urinary NGAL (uNGAL) seems to be more sensitive than plasma NGAL (pNGAL) (19–23), and uNGAL-to-creatinine ratio (UNCR) has been shown to be a specific and sensitive marker for naturally occurring AKI in dogs, although it is also increased in chronic kidney disease (CKD) (24, 25). Urinary gamma-glutamyl transferase (uGGT) is a brush border enzyme located primarily in the metabolically active proximal renal tubule. Higher uGGT to creatinine (uCr) ratios (uGGT/uCr) were found in dogs with AKI and CKD compared with healthy dogs (24, 25).

We conducted an open-label, randomized-controlled trial to evaluate concentrations of plasma creatinine (pCr) and two novel renal biomarkers (pNGAL, uNGAL, UNCR, and uGGT/uCr) in dogs with GDV with and without IV lidocaine. The first objective was to determine renal biomarker concentrations up to 48 h after surgery. The second objective was to compare these biomarkers between dogs with and without IV lidocaine administration. The first hypothesis was that dogs with GDV have increased concentrations of renal biomarkers compared to healthy dogs. The second hypothesis was that dogs receiving IV lidocaine have significantly lower concentrations of renal biomarkers in the post-surgical period compared to dogs not receiving lidocaine, indicating renoprotection.

## Materials and methods

### Study design and settings

Our study was a single center, prospective, randomized, non-blinded, parallel-group clinical trial. It was conducted in the Small Animal Hospital of the Vetsuisse-Faculty of the University of Bern using dogs diagnosed with GDV from August 2017 until September 2018. This study was approved by the Federal Veterinary Service of Switzerland (BE69/17). The patients were treated according to the National Council of Animal Care. Owners signed an informed consent at the time of recruitment. The study is reported in line with the CONSORT statement (26).

### Subjects

All dogs with GDV were eligible for the study. A diagnosis of GDV was based on history and clinical examination findings and was confirmed by abdominal radiographs. Exclusion criteria were known history of kidney disease and administration of nephrotoxic drugs (such as non-steroidal analgesic drugs) within 14 days before inclusion in the study. Some data from the dogs in the non-lidocaine group were used in a previous study (9). Additionally, 18 healthy dogs, which were part of a previous study, served as a healthy control group for pCr, uNGAL and UNCR (23).

### Randomization

Enrolled dogs were randomized to either receive (LIDO group) or not receive (NO-LIDO group) IV lidocaine over a period of 24 h. Randomization was performed by using the permuted block technique (27). A block size of 6 subjects was chosen (3 dogs receiving lidocaine, 3 dogs not receiving lidocaine) and the order of this assignment was random (sealed slips in an envelope).

### Interventions

#### General treatment protocol

All GDV dogs underwent standardized stabilization, anesthesia, surgery, and post-operative monitoring as described in a previous study by the same institution (9). For cardiovascular stabilization, supplemental oxygen and IV isotonic balanced and buffered crystalloids (Plasma-Lyte A^®^, Baxter AG, Switzerland) was administered at the clinician's discretion. Initial analgesia was provided by either IV methadone (0.2 mg/kg; Methadon Streuli^®^, Streuli Pharma AG, Switzerland), or a bolus of fentanyl (5 μg/kg; Fentanyl Curamed^®^, Actavis Switzerland AG, Switzerland) followed by a CRI of fentanyl at a rate of 5 μg/kg/h. In dogs with severe gastric distension, transcutaneous gastrocentesis for gastric decompression with a 14- or 16-gauge needle was performed after initiation of fluid therapy. Blood pressure was evaluated with oscillometry. The administered IV fluid volumes during initial stabilization, and intra- and post-operative periods were recorded throughout the study. To evaluate mentation, a mentation score (28) was performed every 8 h by trained ICU personal.

#### Lidocaine treatment protocol

Dogs assigned to the LIDO group received 2 mg/kg lidocaine (Lidokain 2% Streuli^®^, Streuli Pharma AG, 8730 Uznach, Switzerland) IV, over 15 min parallel to IV fluid therapy but prior to any other intervention. Afterwards, a lidocaine CRI was started at a dose of 50 μg/kg/min, which was continued over 24 h, unless there was a medical reason to stop (e.g., atrioventricular block) or prolong (e.g., sustained ventricular tachycardia) administration. Dogs assigned to the NO-LIDO group did not receive any lidocaine during the entire study period, unless they developed ventricular tachycardia with subsequent cardiovascular compromise. Those dogs were excluded from the study and received antiarrhythmic therapy as needed.

#### Patient data and illness severity scores

Baseline data collected at the time of admission included patient demographics (age, breed, sex, and body weight) and clinical data (mentation, heart rate, respiratory rate, rectal temperature, and systolic blood pressure). An acute patient physiologic and laboratory evaluation (APPLE_fast_) score (28) was compiled at admission (*T*_0_), 24 (*T*_24_) and 48 h (*T*_48_) after surgery. The presence and degree of AKI was evaluated by using the veterinary acute kidney injury (VAKI) staging system as previously described (29). This staging system is based on absolute and relative changes in pCr levels over time (Table 1). For calculation of the VAKI stage, admission (*T*_0_) pCr concentrations were used as a baseline and the highest post-surgery (*T*_24_ or *T*_48_) pCr concentration as the second creatinine concentration for each patient.

**Table 1:** Criteria of different stages of the VAKI scoring system (29).

**VAKI stage**	**Criteria**
Stage 0	Creatinine increase by < 50% from baseline
Stage 1	Creatinine increase by 50–99% from baseline
	OR creatinine increase of 26.5 μmol/L (0.3 mg/dL) from baseline
Stage 2	Creatinine increase by 100–199% from baseline
Stage 3	Creatinine increase of ≥200% from baseline
	OR an absolute creatinine value >354 μmol/L (4.0 mg/dL)

Stage 0, no AKI, stage 1–3 AKI.

#### Sampling and laboratory analysis

Venous blood samples were obtained at admission (prior to any therapeutic intervention, *T*_0_), immediately after surgery (*T*_1_), and at 24 ± 4 h (*T*_24_) and 48 ± 4 h (*T*_48_) post-surgery. At *T*_0_, *T*_24_, and *T*_48_, blood was collected in a 1.3 ml K2-EDTA tube (K2-EDTA Sarstedt AG, Switzerland) and a 9 ml heparin tube (Li-Heparin LH/1.3, Sarstedt AG, Switzerland) for analyses of hematological (Advia^®^ 2120i, Siemens Healthcare Diagnostics AG, Switzerland) and biochemical (Cobas^®^ c501, Roche Diagnostics (Schweiz) AG, 6343 Rotkreuz, Switzerland) variables, and lactate (RAPIDPoint^®^ 500; Siemens Healthcare AG, Switzerland), necessary for calculating the APPLE_fast_ score. An aliquot (0.5 ml) of the heparinized plasma was used for biochemical analyses. The remaining plasma was aliquoted and stored in a −80°C freezer within 1 h of blood collection until batch analyses of pNGAL. At *T*_1_, only a heparin sample was collected for pNGAL immediately after surgery or, for dogs euthanized intraoperatively, immediately prior to euthanasia.

Urine was collected during surgery by cystocentesis or immediately after surgery (*T*_1_) by aseptic catheterization, and at *T*_24_ and *T*_48_ by voided midstream sample. During the daytime, an aliquot of each fresh urine sample was submitted to the laboratory within 2 h for analyses of refractometric USG, dipstick semiquantitative chemistry (Combur 9^®^, Roche Diagnostics (Schweiz) AG, 6343 Rotkreuz, Switzerland), sediment examination, measurement of urine creatinine (uCr) concentration, and uGGT/uCr. The uGGT activity was measured with an enzymatic method (GGT-2, Roche Diagnostics (Schweiz) AG, 6343 Rotkreuz, Switzerland) and uCr was measured using an enzymatic assay (CREP2, Roche Diagnostics (Schweiz) AG, 6343 Rotkreuz, Switzerland) on a biochemistry analyzer (Cobas^®^ c501, Roche Diagnostics (Schweiz) AG, 6343 Rotkreuz, Switzerland). If samples were obtained after laboratory opening hours, refractometric USG and dipstick semiquantitative chemistry were performed immediately. Samples were refrigerated (4°C) overnight, and biochemical and sediment examination was performed the next day. Pyuria was defined as ≥5 leucocytes per high power field and results of all urinary biomarkers were excluded if pyuria was detected.

Aliquots of urine samples were centrifuged twice at 1,500 g for 5 min and the supernatant frozen at −80°C within 1 h after sampling for later batch analysis of uNGAL. Urinary NGAL and pNGAL concentrations were determined using a sandwich ELISA (KIT 036, BioPorto Diagnostics, Gentofte, Denmark) following the manufacturer's instructions using 96-well ELISA plates precoated with a mouse monoclonal antibody against dog NGAL. Briefly, 100 μL of each standard or diluted plasma and urine samples were inoculated into the wells in duplicates. After incubation and washing, another mouse monoclonal antibody labeled with biotin was added to the samples to detect bound NGAL. Horseradish peroxidase-conjugated streptavidin and a color forming substrate were used to develop the assay after another incubation and washing. The absorbance was measured at 450 nm. Samples that showed results out of range were remeasured with higher dilutions until a value in the measurable range was obtained or until a dilution of 1:900 was attained. The UNCR was calculated by dividing uNGAL [ng/dl] and urine creatinine concentration [mmol/l] and by multiplying by 100 (30). Results from blood and urine samples from a previous study evaluating pNGAL, uNGAL, and UNCR in healthy dogs using the same ELISA kit were available for use as a historical healthy control group (23).

### Statistical analyses

Statistical analyses were done using commercial statistical software (MedCalc^®^, Version 20.110, MedCalc statistical software, 8400 Ostend, Belgium). Shapiro-Wilk tests were used to assess normal distribution. As some data were not normally distributed, all data are reported as median with range and/or interquartile range (IQR). Statistical differences of quantitative variables between groups were examined with Mann-Whitney rank sum tests. Significance of differences between the time points within the groups were assessed using Wilcoxon rank sum tests. Categorical variables between groups were analyzed using Chi-squared or Fisher's exact tests. Euthanized and naturally deceased patients were summarized in one non-survivor group for statistical analysis. Statistical significance was defined as *P* ≤ 0.05 and all tests were two-tailed. To counteract the issue of multiple comparisons, Bonferroni corrections were used.

## Results

### Cohort characteristics and outcome

**Figure 1:**
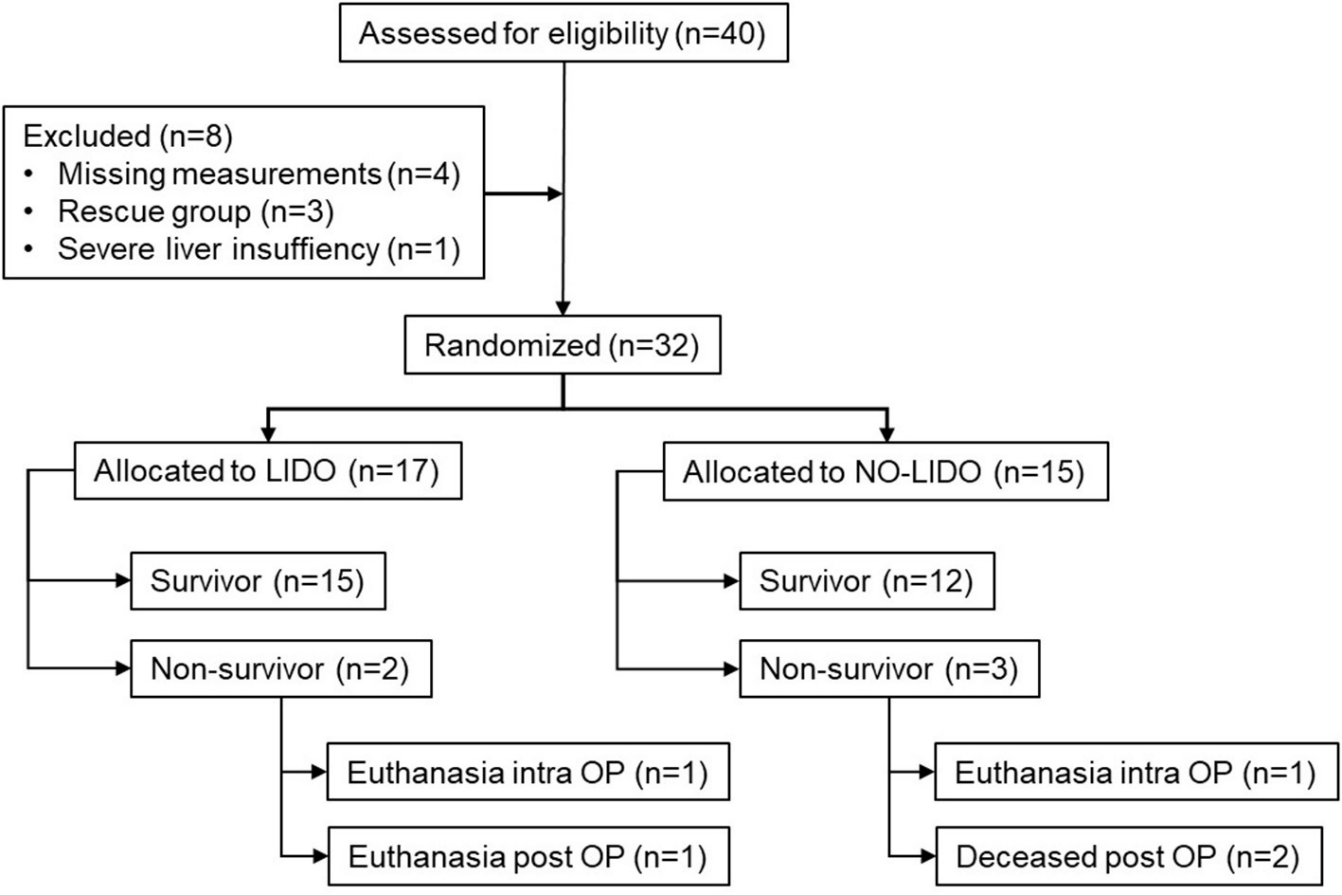
Inclusion and exclusion of 40 dogs presented with GDV into the study cohort. LIDO, GDV dogs treated with lidocaine; NO-LIDO, GDV dogs treated without lidocaine; rescue group, dogs initially enrolled in the NO-LIDO group but needed lidocaine due ventricular tachycardia; intra OP, intra-operatively, post OP, post-operatively.

Forty dogs presented with GDV during the study period. After exclusion of 8 dogs, 32 dogs were enrolled in the study, of which 17 were allocated to the LIDO group and 15 to the NO-LIDO group (Figure 1). Demographic and clinical baseline data are presented in Table 2. The following breeds were represented in the LIDO group: German Shephard (*n* = 3), Great Dane (*n* = 5), and one each of Border Collie, Briard, Bernese Mountain Dog, Dalmatian, Doberman Pinscher, Eurasier, Golden Retriever, Wirehaired Pointing Griffon, Labrador Retriever. In the NO-LIDO group, the following breeds were represented: mixed breed (*n* = 5), St. Bernard (*n* = 2), Weimaraner (*n* = 2), and one each of Great Dane, Golden Retriever, Labrador Retriever, Spanish Mastiff, Newfoundland, and Standard Poodle. The overall mortality was 15.6% (Figure 1), and no significant difference in outcome was found between the two groups (*P* = 0.529). The median volume of crystalloids given between *T*_0_ and *T*_1_ was 91 ml/kg (IQR, 65–133) in the LIDO group and 81 ml/kg (IQR, 64–105) in the NO-LIDO group. The median volume of crystalloids given between *T*_1_ and *T*_24_ was 67 ml/kg (IQR, 53–85) in the LIDO group and 58 ml/kg (IQR, 49–81) in the NO-LIDO group. The median volume of crystalloids given between *T*_24_ and *T*_48_ was 49 ml/kg (IQR, 41–64) in the LIDO group and 48 ml/kg (IQR, 45–52) in the NO-LIDO group. No significant difference in the volume of fluid administered was found at any time point between the groups.

**Table 2:** Demographic and clinical baseline data (median, min–max) in 32 dogs randomized to the LIDO or NO LIDO group.

**Characteristic**	**LIDO (*n* = 17)**	**NO-LIDO (*n* = 15)**	***P*-value**
Age (years)	8.7 (1.5–13.6)	7.6 (2.1–14.5)	0.745
Sex (*n*)			n/a
Female, intact	3	1	
Female, neutered	1	6	
Male, intact	4	4	
Male, neutered	9	4	
Body weight (kg)	36.9 (25.0–80.1)	34.9 (17.3–86.6)	0.763
Mentation score	1 (0–3)	0 (0–3)	0.166
Heart rate (bpm)	140 (72–200)	140 (88–210)	>1
Rectal temperature (°C)	38.5 (37.4–39.8)	38.4 (37.8–39.5)	>1
Respiratory rate (bpm)	40 (24–100)	44 (20–120)	>1
SBP (mmHg)	150 (99–198)	152 (103–194)	>1
APPLE_fast_	22 (15–33)	20 (10–34)	0.375
Transcutaneous gastrocentesis	10	12	0.204
Δ*t* clinicals signs (min)	120 (60–320)	120 (60–540)	0.929
Δ*tT*_0_–surgery (min)	102 (30–159)	82 (46–112)	0.064
Blood lactate (RI: 0.42–2.10 mmol/L)	*T*_0_ 2.92 (1.72–6.80)	*T*_0_ 2.77 (1.35–10.38)	>1
	*T*_1_ 1.48 (0.93–5.38)	*T*_1_ 1.34 (0.55–3.21)	>1
Gastric wall changes	Mild: 10	Mild: 9	0.730
	Moderate: 5	Moderate: 3	
	Severe: 2	Severe: 3	

Mentation score (28): 0, Normal; 1, able to stand unassisted, responsive but dull; 2, can stand only when assisted, responsive but dull; 3, unable to stand, responsive; 4, unable to stand, unresponsive; SBP, systolic blood pressure assessed with oscillometry (SunTech^®^ Vet20™ blood pressure monitor, Morrisville, NC, USA); Δt clinicals signs, duration of clinical signs prior to fluid therapy in minutes; Δt T_0_–surgery; duration of time between admission and surgery in minutes; gastric wall changes according to reference (9).

VAKI staging was only possible in the 27 survivors. All but one patient (96.3%), met the criteria for VAKI stage 0, and one patient (LIDO group) had VAKI stage 1. In this dog, pCr increased by 39 μmol/L between *T*_0_ and *T*_24_, whereby pCr was within the reference range at all time points.

### Plasmatic renal biomarkers

**Figure 2:**
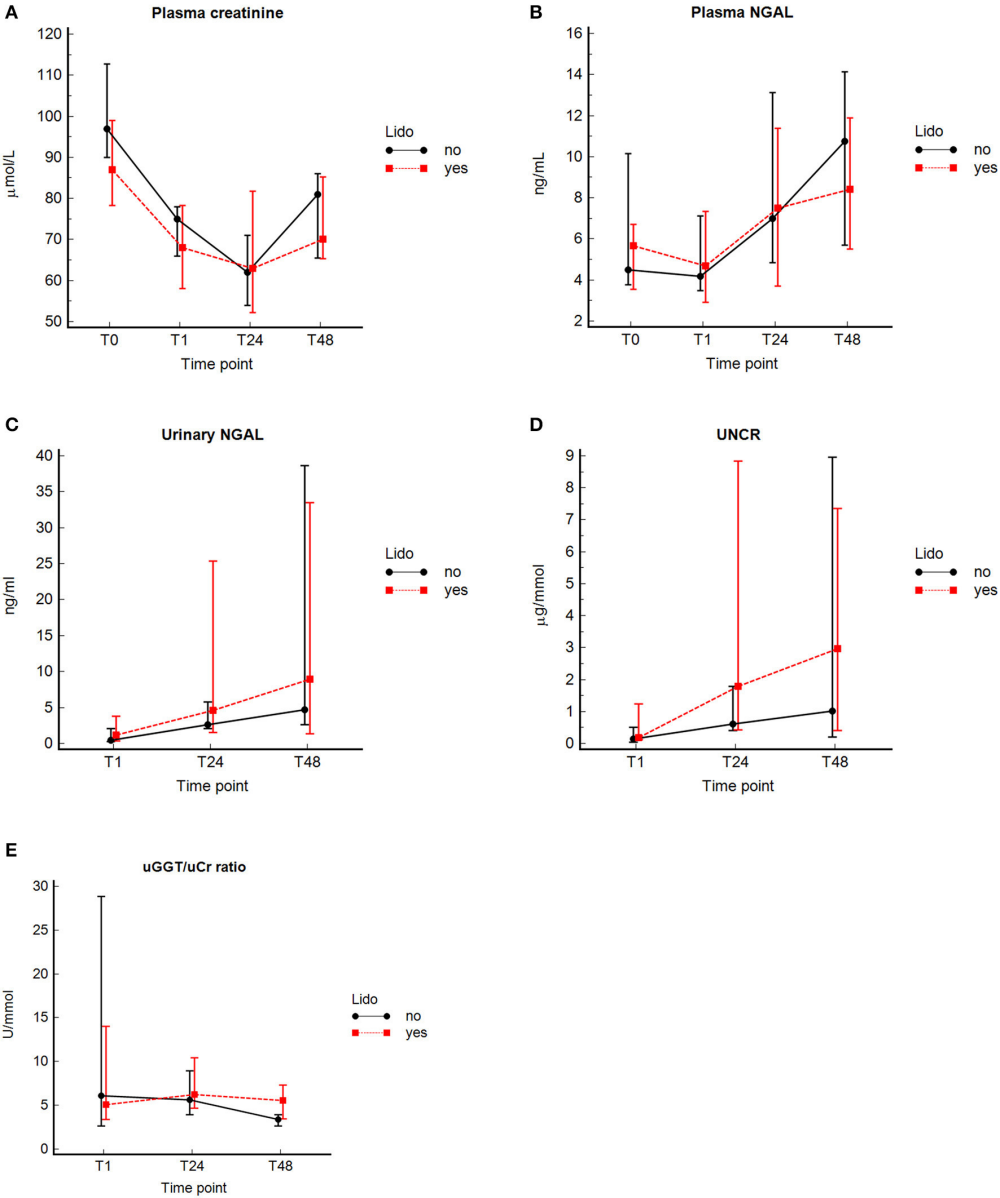
Concentrations (median and interquartile range) of pCr, pNGAL, uNGAL, uGGT/uCr, and UNCR at the different time points in 32 dogs with GDV with and without lidocaine. Median (central value marker-dot) and interquartile range (error bars) of concentrations of plasma creatinine (pCr) **(A)**, plasmatic neutrophil gelatinase-associated lipocalin (pNGAL) **(B)**, urinary NGAL (uNGAL) **(C)**, uNGAL to urine creatinine ratio (UNCR) **(D)**, and urinary gamma-glutamyl transferase to urine creatinine ratio (GGT/uCr) **(E)** in dogs with lidocaine and without lidocaine; *T*_0_, at admission (prior any therapeutic intervention); *T*_1_, immediately after surgery (plasma marker), or during surgery by cystocentesis or immediately after surgery by urinary catheterization (urine marker); and at 24 ± 4 (*T*_24_), and 48 ± 4 h (*T*_48_) post-surgery.

No significant difference in concentrations of pCr and pNGAL was found between the healthy control group and GDV dogs at *T*_0_ (Table 3). At *T*_1_, dogs with GDV had significantly lower pCr and pNGAL (Table 3). No significant difference in pCr and pNGAL was found between GDV dogs with and without lidocaine at any sampling point (Table 4). In the entire population, a significantly higher pCr at *T*_0_ compared with *T*_1_, *T*_24_, and *T*_48_ (*P* < 0.001), and a significant increase of pNGAL between *T*_0_ and *T*_24_ (*P* = 0.006), and between *T*_0_ and *T*_48_ (*P* < 0.001) was found (Table 3; Figure 2).

**Table 3:** Median (IQR) of plasma and urinary renal markers in dogs with GDV and 18 healthy control dogs.

**Variable**	**Time point**	**GDV dogs**	**Historical healthy control group**	***P-*value**
Plasma creatinine (μmol/L)	*T*_0_	95 (82–105)	83.5 (69–94)	0.219
	*T*_1_	69 (60–78)^*^		0.044
	*T*_24_	63 (52–78)^*^		0.015
	*T*_48_	78 (65–87)^*^		0.95
pNGAL (ng/mL)	*T*_0_	5.66 (3.58–7.43)	10.68 (4.60–12.30)	0.061
	*T*_1_	4.42 (3.35–7.30)		0.026
	*T*_24_	7.50 (4.01–11.89)^*^		>1
	*T*_48_	9.86 (5.52–13.92)^*^		>1
uNGAL (ng/mL)	*T*_1_	0.61 (0.30–2.59)	0.2 (0.2–1.25)	0.608
	*T*_24_	2.62 (1.86–10.92)^†^		< 0.001
	*T*_48_	4.79 (1.96–34.97)^†^		< 0.001
UNCR (μg/mmol)	*T*_1_	0.15 (0.09–0.54)	2.11 (1.32–7.45)	< 0.001
	*T*_24_	1.14 (0.41–3.58)^†^		0.153
	*T*_48_	1.34 (0.30–7.42)^†^		0.711
uGGT/uCr (U/mmol)	*T*_1_	5.28 (3.12–14.09)	n/a	n/a
	*T*_24_	6.20 (3.9–9.90)	n/a	n/a
	*T*_48_	3.76 (2.84–6.22)^§^	n/a	n/a

pNGAL, plasma urinary neutrophil gelatinase-associated lipocalin; uNGAL, urinary NGAL; UNCR, urinary NGAL/urine creatinine ratio; uGGT/uCr, urinary gamma-glutamyl transferase to creatinine ratio; T_0_, at admission; T_1_, immediately after surgery (plasma marker) or during surgery by cystocentesis or immediately after surgery by urinary catheterization (urine marker); T_24_, 24 ± 4 h post-surgery; T_48_, 48 ± 4 h post-surgery; ^*^values differing significantly from T_0_ (P < 0.05, Bonferroni corrected); ^†^values differing significantly from T_1_ (P < 0.05, Bonferroni corrected); ^§^values differing significantly from T_24_ (P < 0.05, Bonferroni corrected); P-value, level of significance for comparison between the healthy control group and GDV dogs (Bonferroni corrected).

**Table 4:** Median (IQR) of plasma and urinary renal markers in dogs with GDV treated with lidocaine (LIDO group) or without lidocaine (NO-LIDO group).

**Variable**	**Time point**	**LIDO group**	***n***	**NO-LIDO group**	***n***	***P-*value**
Plasma creatinine (RI: 52–117 μmol/L)	*T*_0_	87 (78–99)	17	97 (90–113)	15	0.236
	*T*_1_	68 (58–78)	17	75 (66–78)	15	>1
	*T*_24_	63 (52–82)	15	62 (54–71)	12	>1
	*T*_48_	70 (65–85)	15	81 (66–86)	12	>1
pNGAL (ng/mL)	*T*_0_	5.66 (3.56–6.72)	17	4.50 (3.78–10.16)	15	>1
	*T*_1_	4.69 (2.90–7.34)	17	4.19 (3.47–7.11)	15	>1
	*T*_24_	7.50 (3.71–11.38)	15	6.98 (4.85–13.14)	12	>1
	*T*_48_	8.41 (5.52–11.88)	15	10.76 (5.68–14.12)	12	>1
uNGAL (ng/mL)	*T*_1_	1.22 (0.39–3.75)	16	0.40 (0.21–2.06)	13	0.287
	*T*_24_	4.57 (1.57–25.41)	12	2.62 (2.06–5.81)	11	>1
	*T*_48_	8.91 (1.34–33.51)	14	4.68 (2.59–38.68)	10	>1
UNCR (μg/mmol)	*T*_1_	0.19 (0.12–1.23)	16	0.10 (0.05–0.49)	13	0.374
	*T*_24_	1.79 (0.43–8.83)	12	0.61 (0.42–1.79)	11	>1
	*T*_48_	2.97 (0.42–7.36)	13	1.03 (0.19–8.96)	10	>1
uGGT/uCr (U/mmol)	*T*_1_	5.06 (3.36–14.04)	14	6.08 (2.66–28.84)	15	>1
	*T*_24_	6.24 (4.70–10.43)	12	5.62 (3.90–8.93)	12	>1
	*T*_48_	5.55 (3.43–7.29)	12	3.38 (2.67–3.96)	12	>1

RI, reference interval; pNGAL, plasma urinary neutrophil gelatinase-associated lipocalin; uNGAL, urinary NGAL; UNCR, urinary NGAL/creatinine ratio; uGGT/uCr ratio, urinary gamma-glutamyl-transferase to creatinine-ratio; T_0_, at admission; T_1_, immediately after surgery (plasma marker) or during surgery by cystocentesis or immediately after surgery by urinary catheterization (urine marker); T_24_, 24 ± 4 h post-surgery; T_48_, 48 ± 4 h post-surgery; P-value, level of significance between the LIDO and NO-LIDO group for each parameter (Bonferroni corrected).

### Urinary renal biomarkers

Compared to controls, uNGAL was significantly higher in dogs with GDV at *T*_24_ and *T*_48_ (Table 3). The UNCR was significantly lower at *T*_1_ in dogs with GDV compared to the healthy control group (Table 3).

No significant difference in uNGAL, UNCR, and uGGT/uCr measurements was found at any time point between GDV dogs with and without lidocaine (Table 4). In the entire population, a significant increase in uNGAL concentrations between *T*_1_ and *T*_24_ (*P* = 0.001) and *T*_1_ and *T*_48_ (*P* < 0.001), and a significant increase of UNCR from *T*_1_ to *T*_24_ (*P* = 0.0015) and from *T*_1_ to *T*_48_ (*P* < 0.001) was found. Concentrations of uGGT/uCr were highest at *T*_24_, and significantly decreased between *T*_24_ and *T*_48_ (*P* < 0.001) (Figure 2, Table 3). For technical reasons, not all of the intended urine samples could be obtained from some patients.

## Discussion

This study evaluated concentrations plasma and urinary renal biomarkers in dogs with GDV and compared the effect of early IV lidocaine therapy followed by a 24-h CRI on these renal biomarkers in a randomized-controlled manner.

The main findings of this study are a significant increase of pNGAL, uNGAL, and UNCR in both groups at *T*_24_ and *T*_48_ compared to *T*_0_ or *T*_1_, respectively, whereas pCr significantly decreased. During the entire study period, pNGAL, uNGAL and UNCR revealed highest values at *T*_48_. As biomarker measurement was not continued beyond 48 h, the duration of increased biomarkers cannot be assessed with our data. However, only uNGAL at *T*_24_ and *T*_48_ was significantly higher than historical healthy controls. Furthermore, a significant change in uGGT/uCr over time was not evident, except a significant decrease between *T*_24_ and *T*_48_ in the NO-LIDO group. Contrary to the authors' hypothesis, no significant differences in urinary and plasmatic renal biomarkers were found between GDV dogs with and without 24-h IV lidocaine therapy.

AKI is described as a complication of GDV in dogs and its incidence may be underestimated in the critical care setting (3, 8, 31). As the kidney receives 20% of the cardiac output, it is vulnerable to global hypoperfusion, and circulatory shock is one potential factor responsible for renal injury (2, 32). Furthermore, general anesthesia, fluid therapy, surgical trauma, blood loss, and repositioning of the stomach leads to congestive nephropathy, IRI, damage induced by pro-inflammatory cytokines, and oxidative stress in dogs with GDV (1, 3, 33). Renal ischemic injury causes damage to the glomerulus, renal tubular cells, and renal vasculature (32). During ischemic AKI, ATP depletion results in cytoskeletal changes in epithelial and endothelial cells, causing disruption of function, and a decrease in glomerular filtration rate. Furthermore, apoptosis and necrosis are major mechanisms of cell death that have important roles in ischemia (34, 35). Two previous studies, assessing the incidence of AKI in dogs with GDV, reported an incidence of 8 and 2.3%, respectively, using serum creatinine concentration as a marker (3, 8). However, serum creatinine concentration is less sensitive than some novel renal biomarkers, among them NGAL, which may result in delayed recognition of renal damage (36). Indeed, NGAL was found to increase 24–72 h earlier than serum creatinine and is considered an early marker for ischemic and nephrotoxic renal injury in both people (14, 18) and dogs (19, 21).

In the present study, the significant increase in pNGAL at 24–48 h after surgery compared to *T*_0_ is probably a sign for renal tubular injury. However, NGAL can be also released from neutrophils and epithelial cells from various tissues (37). Therefore, the origin of the measured pNGAL in dogs in the present study cannot be clearly attributed to the kidney (37). The decrease of pNGAL (and pCr) at *T*_1_ is most likely due to hemodilution after fluid resuscitation. Compared to pNGAL, uNGAL seems to be a more sensitive marker in dogs (19). In a previous study, post-surgery pNGAL did not detect AKI, whereas uNGAL was significantly higher in dogs with AKI at 12 h post-surgery (19). In the present study, uNGAL was significantly increased at *T*_24_ and *T*_48_ comparted to *T*_1_ and was also significantly higher compared to the healthy controls. Increased uNGAL is a consequence of lower reabsorption of uNGAL at the proximal tubulus and/or higher expression of uNGAL mRNA in the tubuli, and indicates renal tubular injury (19, 21, 23, 36, 38).

Although the increase in pNGAL and uNGAL was mild, it is probably a sign of stressed or injured tubular cells, which persisted for at least 48 h after surgery. However, increased release of NGAL of non-renal origin can occur in systemic inflammatory states, such as GDV, and may have contributed to elevated levels found in our study (39). Differentiation between molecular forms of NGAL to determine their origin has been performed in human medicine but such assays are not available for dogs (40). When comparing NGAL concentrations of our study to previous studies in dogs with AKI or chronic kidney disease, values reported in previous studies were higher than those found in our study from dogs with GDV (23, 30, 41). In a previous study, pNGAL concentration < 13.4 ng/ml excluded AKI with a sensitivity of 90%, and pNGAL >64 ng/ml was 100% specific for AKI (23). We found pNGAL >13.4 ng/ml in 6 GDV dogs at *T*_24_, and 11 dogs at *T*_48_, but pNGAL concentrations >64 ng/ml were not found in any dog at any time.

As uGGT does not pass the glomerulus and urinary values are of tubular origin if the glomerulus is intact, uGGT ratio might be a better marker of AKI than uNGAL in states of systemic inflammation like GDV. In humans, measurement of uGGT/uCr resulted in detection of 22% additional patients with AKI compared with plasma creatinine concentration alone in a population of ICU patients (42). Studies in dogs that have evaluated uGGT/uCr as a marker of AKI are inconclusive. In dogs with gentamycin-induced nephrotoxicity, a 2- to 3-fold increase of baseline uGGT/uCr ratio within 24 h indicated early onset of AKI (25). Other recent studies show variable discriminatory ability between dogs with AKI from dogs with chronic kidney disease and healthy controls (24, 43). In addition, the uGGT/uCr ratio has been shown to have significant within-day variations, which may be more pronounced in dogs with AKI compared with healthy dogs (25, 44, 45). In the present study no relevant changes in uGGT/uCr over time were found. One reason may be a lower sensitivity of uGGT compared to NGAL-based markers. Furthermore, the inflammatory component may have led to a non-renal increase in NGAL.

The incidence of AKI in dogs with GDV using the VAKI staging system varied in our study depending on the time point used as a baseline. We used pCr at *T*_0_ as the baseline value (before fluid resuscitation), resulting in a diagnosis of AKI in only one dog (3.7%, VAKI stage 1). If assessed using pCr at *T*_24_ as the baseline (24 h after fluid resuscitation and hemodilution) and *T*_48_ as the second time point, the incidence VAKI stage 1 was 14.8% (4–3 dogs in the LIDO group, 1 dog in the NO-LIDO-group). In general, comparing biomarkers of assumed parenchymal kidney injury against a creatinine-based reference method that imperfectly estimates functional kidney impairment is problematic (46). Indeed, dilution or concentration of pCr in fluid-loaded or hemoconcentrated patients may lead to under- or overestimation of AKI assessed by creatinine-based criteria. At time *T*_0_, most dogs were probably hemoconcentrated, and at *T*_1_ they were hemodiluted due to aggressive fluid resuscitation. Therefore, VAKI is of questionable suitability for the diagnosis of AKI in dogs with GDV. Moreover, other creatinine-based AKI classifications systems, such as Risk–Injury–Failure–Loss–Endstage renal disease (RIFLE) and Acute Kidney Injury Network (AKIN) are likely of no greater utility (47). Regarding the use of ratios of urinary biomarkers to creatinine, uCr excretion is probably not constant in dogs with an unsteady hemodynamic state like GDV dogs. Under non-steady-state conditions, such as shock and subsequent high fluid rates, uCr excretion changes over time. Unless the considered biomarker behaves exactly like creatinine (i.e., filtered, secreted to some extent, normally not absorbed), the ratio is influenced by differences in uCr excretion (46). Normalization of biomarkers by uCr ratios may, therefore, result in both underestimation and overestimation of the biomarker excretion depending on the clinical context (46).

Lidocaine is a well-known local anesthetic and antiarrhythmic drug. Lidocaine CRI further prevents the intraoperative nociceptive response in dogs due to its analgesic potential, thereby reducing the intraoperative use of opioids (i.e., fentanyl) without causing clinically significant hemodynamic instability (48). In addition, lidocaine exhibits cytoprotective effects, as it exhibits antioxidant actions, such as reduction in the formation of reactive oxygen species and lipid peroxidation, and anti-inflammatory actions (10, 11). Suggested mechanisms for these effects include the inhibition of transcellular Na^+^/Ca^2+^ exchange and subsequent reduction of intracellular Ca^2+^ accumulation during ischemia, scavenging of radicals, diminished release of superoxide from granulocytes, and decreased immune cell activation, migration into ischemic tissue, and subsequent cell death and endothelial damage (3, 10). In rats with diabetic nephropathy, renoprotective effects such as decreased inflammation and oxidative stress, and improved oxygenation and renal functions were found (49). Experimental studies found gastric and cardiac organoprotection by IV lidocaine in rats and dogs (12, 50, 51). Bruchim et al. found that in dogs with spontaneous GDV, early IV lidocaine bolus followed by a 24-h CRI post-admission significantly decreased the occurrence of AKI, cardiac arrhythmias, and the length of hospitalization (3). However, these results cannot be directly compared with our results because the study designs differed and only one dog (LIDO group) developed VAKI stage 1 in our study population. Nevertheless, we did not find any significant difference in plasmatic or urinary renal biomarkers between GDV dogs with and without lidocaine treatment and we could not confirm any renoprotective potential based on our study results.

There are some limitations in this study. The cohort size of dogs with GDV was small, which could entail type 2 errors. Because the study ended after 48 h, an increase in pCr or other renal biomarkers shortly after the end of the study cannot be excluded. Furthermore, institutional reference ranges for NGAL (pNGAL, uNGAL, UNCR and uGGT/uCr) were not available, and results in GDV dogs from the present study were compared to values from a small historical healthy control group from a study performed in 2014 (except for uGGT/uCr) (30). Since the ELISA kit used in the control group was from the same manufacturer but not from the same LOT number, the comparability of the renal biomarker values between GDV dogs and a historical control group is questionable. In addition, dogs of the control group were neither age nor breed matched to the GDV dogs. Because of possible age- and breed-related differences in the concentrations of renal biomarkers or the risk of developing AKI, matching on breed and age would have been advantageous to exclude the influence of these risk factors (52).

## Conclusion

The results of this study show that pNGAL, uNGAL, and UNCR significantly increased during the 48-h period post-surgery, whereas pCr did not. This suggests stressed or injured tubular cells and a potentially higher incidence of AKI in dogs with GDV than previously described, which is likely underestimated when pCr concentrations alone are evaluated. Administration of lidocaine did not affect concentrations of renal markers in dogs with GDV.

## Data availability statement

The original contributions presented in the study are included in the article/supplementary material, further inquiries can be directed to the corresponding author.

## Ethics statement

The animal study was reviewed and approved by Animal Experiment Committee of the Swiss Federal Veterinary Office (registration number BE 69/17). Written informed consent was obtained from the owners for the participation of their animals in this study.

## Author contributions

AL: sample collection, study design, laboratory analyses, data analysis, and preparation of the manuscript. AB: sample collection and data analysis. TF and SS: preparation of the manuscript. LP and EM: laboratory analyses and preparation of the manuscript. K-NA: study design, sample collection, data analysis, and preparation of the manuscript. All authors contributed to, read, and approved the final manuscript.

## References

[1] Monnet E. Gastric dilatation-volvulus syndrome in dogs. Vet Clin North Am Small Anim Pract. (2003) 33:987–1005. 10.1016/S0195-5616(03)00059-7 14552158

[2] Sharp CR Rozanski EA. Cardiovascular and systemic effects of gastric dilatation and volvulus in dogs. Top Companion Anim Med. (2014) 29:67–70. 10.1053/j.tcam.2014.09.007 25496923

[3] Bruchim Y Itay S Shira BH Kelmer E Sigal Y Itamar A. Evaluation of lidocaine treatment on frequency of cardiac arrhythmias, acute kidney injury, and hospitalization time in dogs with gastric dilatation volvulus. J Vet Emerg Crit Care. (2012) 22:419–27. 10.1111/j.1476-4431.2012.00779.x 22805421

[4] Muir W. Gastric dilatation-volvulus in the dog, with emphasis on cardiac arrhythmias. J Am Vet Med Assoc. (1982) 180:739–42. 7085452

[5] Muir W Lipowitz A. Cardiac dysrhythmias associated with gastric dilatation-volvulus in the dog. J Am Vet Med Assoc. (1978) 172:683–9. 640931

[6] Muir WW. Acid-base and electrolyte disturbances in dogs with gastric dilatation-volvulus. J Am Vet Med Assoc. (1982) 181:229–31. 7107499

[7] Uhrikova I Machackova K Rauserova-Lexmaulova L. Disseminated intravascular coagulation in dogs with gastric dilatation-volvulus syndrome. Veterinární *Medicina*. (2013) 58:587–90. 10.17221/7141-VETMED

[8] Buber T Saragusty J Ranen E Epstein A Bdolah-Abram T Bruchim Y. Evaluation of lidocaine treatment and risk factors for death associated with gastric dilatation and volvulus in dogs: 112 cases (1997–2005). J Am Vet Med Assoc. (2007) 230:1334–9. 10.2460/javma.230.9.1334 17472559

[9] Brunner A Schuller S Hettlich B Marti E Lehmann A Peters LM. Kinetics of plasma cytokines, angiopoietin-2, and C-reactive protein in dogs with gastric dilatation volvulus. Front Vet Sci. (2021) 8:652479. 10.3389/fvets.2021.652479 34222394 PMC8242176

[10] Cassutto B Gfeller R. Use of intravenous lidocaine to prevent reperfusion injury and subsequent multiple organ dysfunction syndrome. J Vet Emerg Crit Care. (2003) 13:138–48. 10.1046/j.1435-6935.2003.00080.x

[11] Cassuto J Sinclair R Bonderovic M. Anti-inflammatory properties of local anesthetics and their present and potential clinical implications. Acta Anaesthesiol Scand. (2006) 50:265–82. 10.1111/j.1399-6576.2006.00936.x 16480459

[12] Pfeiffer CJ Keith JC Cho CH DeRolf S Pfeiffer DC Misra HP. Gastric and cardiac organoprotection by lidocaine. Acta Physiol Hung. (1989) 73:129–36. 2596305

[13] Kjeldsen L Bjerrum OW Hovgaard D Johnsen AH Sehested M Borregaard N. Human neutrophil gelatinase: a marker for circulating blood neutrophils. Purification and quantitation by enzyme linked immunosorbent assay. Eur J Haematol. (1992) 49:180–91. 10.1111/j.1600-0609.1992.tb00045.x 1464361

[14] Mishra J Dent C Tarabishi R Mitsnefes MM Ma Q Kelly C. Neutrophil gelatinase-associated lipocalin (NGAL) as a biomarker for acute renal injury after cardiac surgery. Lancet. (2005) 365:1231–8. 10.1016/S0140-6736(05)74811-X 15811456

[15] Mishra J Ma Q Prada A Mitsnefes M Zahedi K Yang J. Identification of neutrophil gelatinase-associated lipocalin as a novel early urinary biomarker for ischemic renal injury. J Am Soc Nephrol. (2003) 14:2534–43. 10.1097/01.ASN.0000088027.54400.C6 14514731

[16] Mori K Nakao K. Neutrophil gelatinase-associated lipocalin as the real-time indicator of active kidney damage. Kidney Int. (2007) 71:967–70. 10.1038/sj.ki.5002165 17342180

[17] Wagener G Jan M Kim M Mori K Barasch JM Sladen RN. Association between increases in urinary neutrophil gelatinase-associated lipocalin and acute renal dysfunction after adult cardiac surgery. Anesthesiology. (2006) 105:485–91. 10.1097/00000542-200609000-00011 16931980

[18] Haase M Haase-Fielitz A Bellomo R Mertens PR. Neutrophil gelatinase-associated lipocalin as a marker of acute renal disease. Curr Opin Hematol. (2011) 18:11–8. 10.1097/MOH.0b013e3283411517 21102325

[19] Lee YJ Hu YY Lin YS Chang CT Lin FY Wong ML. Urine neutrophil gelatinase-associated lipocalin (NGAL) as a biomarker for acute canine kidney injury. BMC Vet Res. (2012) 8:248. 10.1186/1746-6148-8-248 23270335 PMC3549924

[20] Westhuyzen J Endre ZH Reece G Reith DM Saltissi D Morgan TJ. Measurement of tubular enzymuria facilitates early detection of acute renal impairment in the intensive care unit. Nephrol Dial Transplant. (2003) 18:543–51. 10.1093/ndt/18.3.543 12584277

[21] Segev G Palm C LeRoy B Cowgill LD Westropp JL. Evaluation of neutrophil gelatinase-associated lipocalin as a marker of kidney injury in dogs. J Vet Intern Med. (2013) 27:1362–7. 10.1111/jvim.12180 24020513

[22] Hsu WL Lin YS Hu YY Wong ML Lin FY Lee YJ. Neutrophil gelatinase-associated lipocalin in dogs with naturally occurring renal diseases. J Vet Intern Med. (2014) 28:437–42. 10.1111/jvim.12288 24417186 PMC4858015

[23] Steinbach S Weis J Schweighauser A Francey T Neiger R. Plasma and urine neutrophil gelatinase-associated lipocalin (NGAL) in dogs with acute kidney injury or chronic kidney disease. J Vet Intern Med. (2014) 28:264–9. 10.1111/jvim.12282 24417647 PMC4857964

[24] Lippi I Perondi F Meucci V Bruno B Gazzano V Guidi G. Clinical utility of urine kidney injury molecule-1 (KIM-1) and gamma-glutamyl transferase (GGT) in the diagnosis of canine acute kidney injury. Vet Res Commun. (2018) 42:95–100. 10.1007/s11259-018-9711-7 29427053

[25] Rivers BJ Walter PA O'Brien TD King VL Polzin DJ. Evaluation of urine gamma-glutamyl transpeptidase-to-creatinine ratio as a diagnostic tool in an experimental model of aminoglycoside-induced acute renal failure in the dog. J Am Anim Hosp Assoc. (1996) 32:323–36. 10.5326/15473317-32-4-323 8784723

[26] Schulz KF Altman DG Moher D Group C CONSORT. 2010 statement: updated guidelines for reporting parallel group randomised trials. BMJ. (2010) 340:c332. 10.1136/bmj.c332 20332509 PMC2844940

[27] Broglio K. Randomization in clinical trials: permuted blocks and stratification. JAMA. (2018) 319:2223–4. 10.1001/jama.2018.6360 29872845

[28] Hayes G Mathews K Doig G Kruth S Boston S Nykamp S. The acute patient physiologic and laboratory evaluation (APPLE) score: a severity of illness stratification system for hospitalized dogs. J Vet Intern Med. (2010) 24:1034–47. 10.1111/j.1939-1676.2010.0552.x 20629945

[29] Thoen ME Kerl ME. Characterization of acute kidney injury in hospitalized dogs and evaluation of a veterinary acute kidney injury staging system. J Vet Emerg Crit Care. (2011) 21:648–57. 10.1111/j.1476-4431.2011.00689.x 22316258

[30] Cobrin AR Blois SL Abrams-Ogg AC Kruth SA Dewey C Holowaychuk MK. Neutrophil gelatinase-associated lipocalin in dogs with chronic kidney disease, carcinoma, lymphoma and endotoxaemia. J Small Anim Pract. (2016) 57:291–8. 10.1111/jsap.12481 27112380

[31] Torrente C Molina C Bosch L Costa-Farre C. Transient distal renal tubular acidosis in a dog with gastric-dilatation-volvulus. Can Vet J. (2019) 60:174–8. 30705453 PMC6340260

[32] Bonventre JV Yang L. Cellular pathophysiology of ischemic acute kidney injury. J Clin Invest. (2011) 121:4210–21. 10.1172/JCI45161 22045571 PMC3204829

[33] D'Marco L. Congestive nephropathy. Int J Environ Res Public Health. (2022) 19:2499. 10.3390/ijerph19052499 35270191 PMC8909002

[34] Sharfuddin AA Molitoris BA. Pathophysiology of ischemic acute kidney injury. Nat Rev Nephrol. (2011) 7:189–200. 10.1038/nrneph.2011.16 21364518

[35] Verma SK Molitoris BA. Renal endothelial injury and microvascular dysfunction in acute kidney injury. Semin Nephrol. (2015) 35:96–107. 10.1016/j.semnephrol.2015.01.010 25795503 PMC4476528

[36] Palm CA Segev G Cowgill LD LeRoy BE Kowalkowski KL Kanakubo K. Urinary neutrophil gelatinase-associated lipocalin as a marker for identification of acute kidney injury and recovery in dogs with gentamicin-induced nephrotoxicity. J Vet Intern Med. (2016) 30:200–5. 10.1111/jvim.13819 26725776 PMC4913669

[37] Singer E Marko L Paragas N Barasch J Dragun D Muller DN. Neutrophil gelatinase-associated lipocalin: pathophysiology and clinical applications. Acta Physiol. (2013) 207:663–72. 10.1111/apha.12054 23375078 PMC3979296

[38] Zhou X Ma B Lin Z Qu Z Huo Y Wang J. Evaluation of the usefulness of novel biomarkers for drug-induced acute kidney injury in beagle dogs. Toxicol Appl Pharmacol. (2014) 280:30–5. 10.1016/j.taap.2014.07.002 25034533

[39] Monari E Troia R Magna L Gruarin M Grisetti C Fernandez M. Urine neutrophil gelatinase-associated lipocalin to diagnose and characterize acute kidney injury in dogs. J Vet Intern Med. (2020) 34:176–85. 10.1111/jvim.15645 31705606 PMC6979095

[40] Cai L Rubin J Han W Venge P Xu S. The origin of multiple molecular forms in urine of HNL/NGAL. Clin J Am Soc Nephrol. (2010) 5:2229–35. 10.2215/CJN.00980110 20829422 PMC2994084

[41] Scheemaeker S Meyer E Schoeman JP Defauw P Duchateau L Daminet S. Urinary neutrophil gelatinase-associated lipocalin as an early biomarker for acute kidney injury in dogs. Vet J. (2020) 255:105423. 10.1016/j.tvjl.2019.105423 31982082

[42] Blasco V Wiramus S Textoris J Antonini F Bechis C Albanese J. Monitoring of plasma creatinine and urinary gamma-glutamyl transpeptidase improves detection of acute kidney injury by more than 20%. Crit Care Med. (2011) 39:52–6. 10.1097/CCM.0b013e3181fa431a 21178528

[43] Nivy R Avital Y Aroch I Segev G. Utility of urinary alkaline phosphatase and gamma-glutamyl transpeptidase in diagnosing acute kidney injury in dogs. Vet J. (2017) 220:43–7. 10.1016/j.tvjl.2016.12.010 28190493

[44] Gossett KA Turnwald GH Kearney MT Greco DS Cleghorn B. Evaluation of gamma-glutamyl transpeptidase-to-creatinine ratio from spot samples of urine supernatant, as an indicator of urinary enzyme excretion in dogs. Am J Vet Res. (1987) 48:455–7. 2882712

[45] Heiene R Biewenga W Koeman J. Urinary alkaline phosphatase and 7-glutamyl transferase as indicators of acute renal damage in dogs. J Small Anim Pract. (1991) 32:521–4. 10.1111/j.1748-5827.1991.tb00871.x

[46] Waikar SS Sabbisetti VS Bonventre JV. Normalization of urinary biomarkers to creatinine during changes in glomerular filtration rate. Kidney Int. (2010) 78:486–94. 10.1038/ki.2010.165 20555318 PMC3025699

[47] Cruz DN Ricci Z Ronco C. Clinical review: RIFLE and AKIN—time for reappraisal. Crit Care. (2009) 13:211. 10.1186/cc7759 19638179 PMC2717405

[48] Ortega M Cruz I. Evaluation of a constant rate infusion of lidocaine for balanced anesthesia in dogs undergoing surgery. Can Vet J. (2011) 52:856–60. 22294791 PMC3135028

[49] Zhang HQ Fang BJ Zhang QZ Ji X-B Chen L. Renoprotective effect of lidocaine on streptozotocin-induced diabetic nephropathy. Int J Clin Exp Med. (2016) 9:14254–9.

[50] Schaub RG Stewart G Strong M Ruotolo R Lemoie G. Reduction of ischemic myocardial damage in the dog by lidocaine infusion. Am J Pathol. (1977) 87:399–414. 851172 PMC2032039

[51] van Emous JG Nederhoff MG Ruigrok TJ van Echteld CJ. The role of the Na+ channel in the accumulation of intracellular Na+ during myocardial ischemia: consequences for post-ischemic recovery. J Mol Cell Cardiol. (1997) 29:85–96. 10.1006/jmcc.1996.0254 9040024

[52] Coyne M Szlosek D Clements C McCrann D Olavessen L. Association between breed and renal biomarkers of glomerular filtration rate in dogs. Vet Rec. (2020) 187:e82. 10.1136/vr.105733 32611706 PMC7799420

