# Erratum: Concentrations and kinetics of renal biomarkers in dogs with gastric dilatation-volvulus with and without 24-h intravenous lidocaine

**DOI:** 10.3389/fvets.2023.1175341

**Published:** 2023-04-20

**Authors:** Frontiers Production Office

**Affiliations:** Frontiers Media SA, Lausanne, Switzerland

**Keywords:** AKI, renal biomarker, canine gastric torsion, ischemia-reperfusion, organoprotection, lidocaine

An omission in the funding section of the original article was made in error. The following sentence has been added: “Open access funding was provided by the University of Bern.”

The original article has been updated.

